# Determination of 1,3-Diphenylguanidine, 1,3-Di-*o*-tolylguanidine, and 1,2,3-Triphenylguanidine in Human Urine Using Liquid Chromatography-Tandem Mass Spectrometry

**DOI:** 10.1021/acs.est.3c00412

**Published:** 2023-06-08

**Authors:** Zhong-Min Li, Kurunthachalam Kannan

**Affiliations:** † Department of Pediatrics, New York University Grossman School of Medicine, New York, New York 10016, United States; ‡ Department of Environmental Medicine, New York University Grossman School of Medicine, New York, New York 10016, United States

**Keywords:** 1,3-diphenylguanidine, 1,3-di-*o*-tolylguanidine, 1,2,3-triphenylguanidine, urine, human exposure, rubber additive

## Abstract

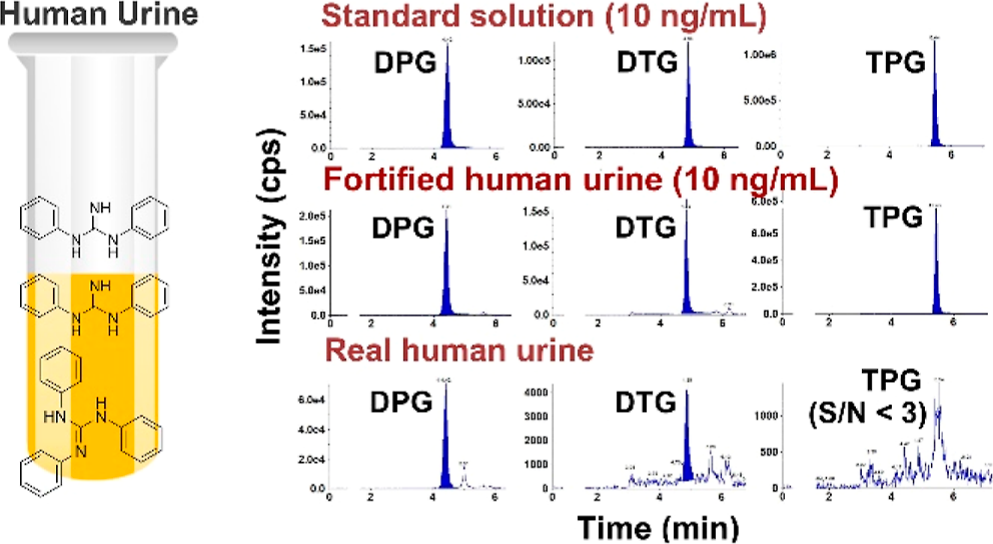

1,3-Diphenylguanidine (DPG), 1,3-di-*o*-tolylguanidine (DTG), and 1,2,3-triphenylguanidine (TPG) are rubber additives widely present in the indoor environment. Nevertheless, little is known about their human exposure. We developed a method for the quantification of DPG, DTG, and TPG in human urine, using high-performance liquid chromatography-tandem mass spectrometry. The quantitative analysis of target analytes at parts-per-trillion levels in urine was optimized using hydrophilic–lipophilic balanced solid-phase extraction and isotopic dilution. The method limits of detection and quantification were in the range of 0.002–0.02 and 0.005–0.05 ng/mL, respectively. The recoveries of all analytes in human urine fortified at 1, 5, 10, and 20 ng/mL concentrations were in the range of 75.3–111%, with standard deviations of 0.7–4%. The repeated measurement of similarly fortified human urine yielded intra-day and inter-day variations of 0.47–3.90 and 0.66–3.76%, respectively. The validated method was applied in the measurement of DPG, DTG, and TPG in real human urine samples, which revealed the occurrence of DPG in children’s urine samples (*n* = 15) with a detection frequency of 73% and at a median concentration of 0.05 ng/mL. DPG was found in 20% of adults’ urine samples (*n* = 20).

## Abstract 2

Diphenylguanidine, a tire wear product, is present widely in human urine.

## 1 Introduction

1,3-Diphenylguanidine (DPG), 1,3-di-*o*-tolylguanidine (DTG), and 1,2,3-triphenylguanidine (TPG) are synthetic chemicals typically used in vulcanization of rubber^1^ and have been found in rubber products such as tires, shoes, furniture, gloves, electric/electronic products, as well as high-density polyethylene-based materials.^2−6^ The aggregate annual production volumes of DPG and DTG in 2019 in the United States were 454–4540 and 61 tons, respectively (https://chemview.epa.gov/chemview/). With the aging of tires and other rubber products, these compounds are gradually released into the environment, leading to potential human exposure. The estimated per capita emission of tire wear particles (TWPs) ranged from 0.23 to 4.7 kg/year globally.^7^ Considerable attention has been paid recently on the safety of TWPs since the finding of 6PPD-Q, a derivative of *N*-(1,3-dimethylbutyl)-*N*′-phenyl-*p*-phenylenediamine (6PPD; a rubber additive), as the cause for acute mortality of coho salmon (*Oncorhynchus kisutch*) in the United States Pacific Northwest.^8−10^

Few studies have reported the occurrence of DPG, DTG, and TPG in the environment. DPG and DTG were found in surface and tap water from several countries at concentrations generally below 1 ng/mL.^4,11−16^ DPG, DTG, and TPG were found in indoor dust collected from various countries.^17−19^ In our earlier study, we found DPG, DTG, and TPG in 100, 62, and 76%, respectively, of the house dust samples collected from 11 countries (*n* = 332) at median concentrations of 140, 2.3, and 0.9 ng/g, respectively.^19^ These findings suggest potential for human exposure to DPG, DTG, and TPG through dust ingestion and drinking water consumption.

Toxicological studies have suggested reproductive toxicity,^20^ neurotoxicity,^17^ and endocrine-disrupting potential of DPG.^17^ Furthermore, dermal exposure to DPG, DTG, and TPG has been linked to allergic contact dermatitis.^6,21−23^ Laboratory studies showed that following oral administration of rats to DPG, this compound was rapidly absorbed, distributed, metabolized, and excreted in urine and feces as both the parent compound and metabolites, with a biological half-life of ∼10 h.^24^ However, the metabolites of DPG in urine remain unidentified. Besides, the metabolism of DTG and TPG has not been investigated. Recently, a Chinese human biomonitoring study reported the occurrence of DPG in maternal and cord serum at median concentrations of 1.70 and 0.35 ng/mL, respectively.^25^

The United States Environmental Protection Agency identified DPG as a chemical marker for quantification of the magnitude of indoor dust ingestion in children.^26^ This requires the assessment of internal exposure dose of DPG. Urine is a preferred matrix to assess internal exposure doses of DPG, DTG, and TPG, but to date, a method to measure these chemicals in urine is not available.

The goal of this study was to develop and validate a method for the determination of DPG, DTG, and TPG in human urine using isotope dilution high-performance liquid chromatography-tandem mass spectrometry (HPLC–MS/MS). The method involving solid-phase extraction (SPE) was optimized to remove matrix components and improve sensitivity. The method was validated through the evaluation of accuracy, precision, matrix effect, and sensitivity. Finally, the method was applied in the measurement of DPG, DTG, and TPG in 20 adults’ and 15 children’s urine samples.

## 2 Materials and Methods

### 2.1 Chemicals and Reagents

**Figure 1:**
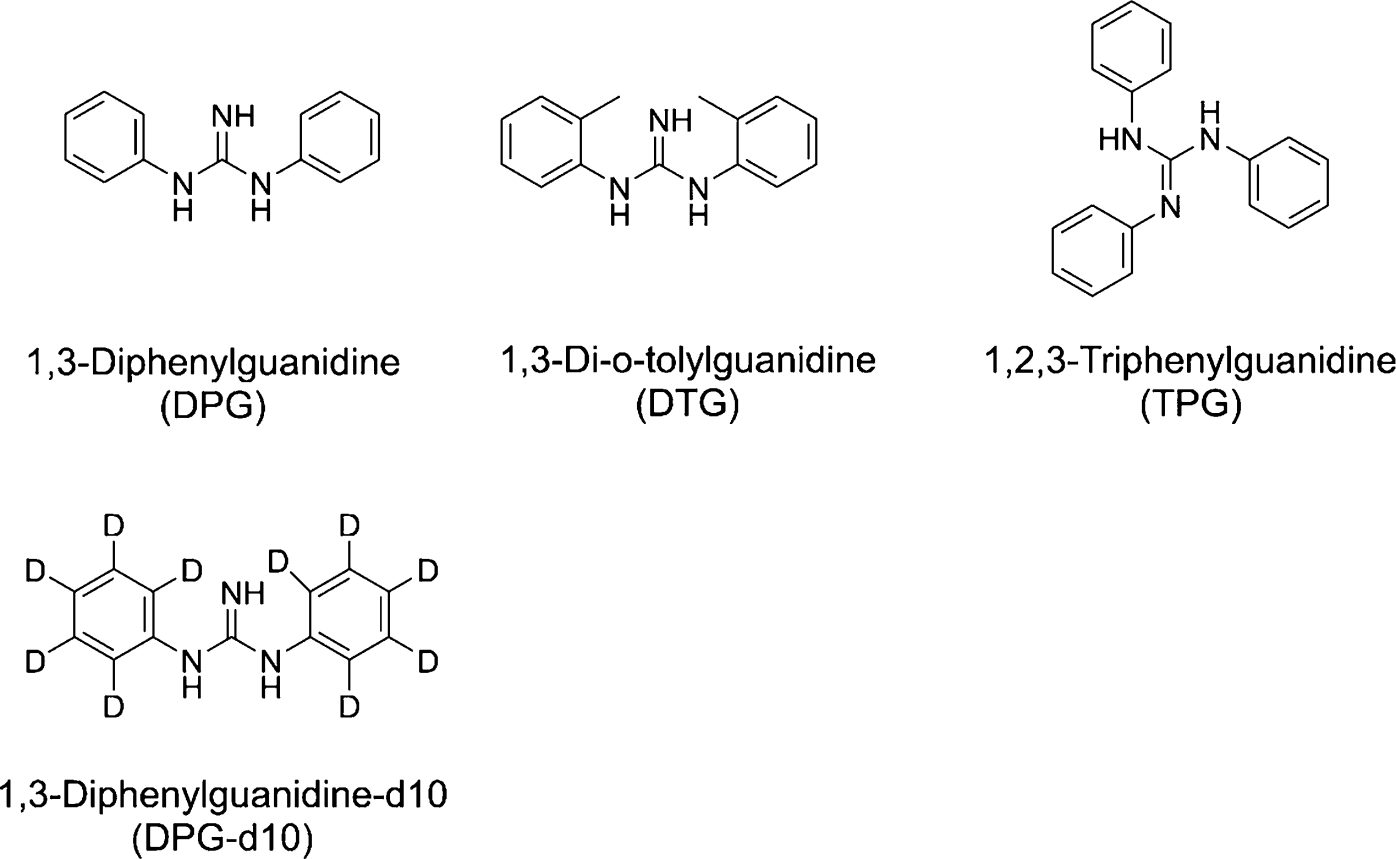
Molecular structures of DPG (CAS# 102-06-7), DTG (CAS# 97-39-2), TPG (CAS# 101-01-9), and the isotopically labeled DPG (DPG-d_10_) investigated in this study.

The chemical structures of the analytes are given in Figure 1. Analytical standards of DPG, DTG, and TPG with purities ≥95% were purchased from Millipore-Sigma (St. Louis, MO, USA). Isotopically labeled DPG (DPG-d_10_; purity ≥97%) was obtained from Toronto Research Chemicals (Toronto, ON, Canada). Ammonium formate (97%; analytical grade) and formic acid (88%; analytical grade) were purchased from Millipore-Sigma (St. Louis, MO, USA). Methanol (MeOH) and water of LC–MS grade were obtained from Fisher Scientific (Waltham, MA, USA). OasisⓇ hydrophilic–lipophilic balanced (HLB) cartridges (60 mg/3 mL) were purchased from Waters Corporation (Milford, MA, USA). Synthetic urine was purchased from Cerilliant (Round Rock, TX, USA). Individual stock solutions of all analytes and DPG-d_10_ were prepared in MeOH at 1 mg/mL. Working solutions were diluted from stock solutions using MeOH.

### 2.2 Urine Samples

Archived human urine samples (*n* = 35) were analyzed using the method developed in this study, to assess the feasibility of the method. Twenty spot urine samples collected randomly from 20 adult volunteers (age: 20–55 years) from New York City during May–June 2022 were analyzed. Samples were collected directly into 50 mL polypropylene (PP) tubes and stored at −20 °C until analysis.^27^ Fifteen children’s spot urine samples (age: 5–7 years) collected from New York City during 2013–2017, and archived in our laboratory at −80 °C, were analyzed. All of the urine samples were deidentified and therefore fell under the exempt category of the New York University Institutional Review Board.

### 2.3 Sample Extraction

Pooled human urine fortified with target analytes at 1, 5, 10, and 20 ng/mL were used for method development and validation. A 500 μL aliquot of the urine sample was transferred into a 15 mL PP tube. The isotopically labeled internal standard (2.5 ng of DPG-d_10_) was spiked into each urine sample, which was vortexed and kept at room temperature for 30 min. The sample was subsequently loaded onto an Oasis HLB cartridge (60 mg/3 mL) that had been preconditioned with sequential elution of 3 mL of MeOH and 3 mL of water. The cartridge was then washed with 3 mL of water/MeOH (95:5, v/v) and dried under vacuum for 5 min to remove residual moisture. The analytes were eluted into a clean 15 mL PP tube using 3 mL of MeOH. The elute was evaporated to near-dryness under a gentle nitrogen stream at 40 °C. The residue was reconstituted in 250 μL of MeOH, vortexed, and transferred into a glass vial.

### 2.4 High-Performance Liquid Chromatography-Tandem Mass Spectrometry

Chromatographic separation of target analytes was accomplished using an ExionLC HPLC (SCIEX, Redwood City, CA, USA) fitted with an Ultra AQ C18 analytical column (3 μm, 100 × 2.1 mm; Restek, Bellefonte, PA, USA), which was serially connected to a BetaSil C18 Javelin guard column (5 μm, 20 × 2.1 mm; Thermo Fisher Scientific, Waltham, MA, USA). The mobile phases were as follows: (A) 5 mM ammonium formate and (B) MeOH each containing 0.1% formic acid (v/v), maintained at a flow rate of 0.3 mL/min. The binary mobile phase flow started from 10% B held for 0.5 min and increased to 90% B over 5 min, which was maintained for 2 min, then returned to the initial condition in 0.5 min, and equilibrated for 2 min prior to the next injection. The HPLC column and the autosampler were maintained at 35 °C and 15 °C, respectively. The injection volume was 2 μL.

The detection of target analytes was performed on an ABSciex 5500+ Q-Trap MS/MS (Framingham, MA, USA) operated in the multiple reaction monitoring (MRM) positive-ionization mode. The MRM parameters including declustering potential (DP), collision energy (CE), entrance potential (EP), collision cell exit potential (CXP), and dwell time were optimized through infusion of a standard solution (at 100 ng/mL) of target analytes (Table 1). The instrument-specific parameters were as follows: ionspray voltage 5.5 kV, ion-source temperature 550 °C, curtain gas flow rate 20 psi, collision gas flow rate 9 psi, ion-source gas 1 flow rate 70 psi, and ion-source gas 2 flow rate 60 psi. Acquisition of data was performed using Analyst software (v1.7.2; ABSciex, Framingham, MA, USA).

**Table 1:** Optimized MRM Parameters of DPG, DTG, TPG, and the Isotope-Labeled DPG (DPG-d_10_) Included in This Study^a^

analytes	Q1 (*m*/*z*)	Q3 (*m*/*z*)	DP (V)	CE (V)	EP (V)	CXP (V)	dwell time (ms)
DPG	212	119	100	31	10	10	80
		*77*^b^	*80*	*51*	*10*	*12*	*80*
DTG	240	133	98	32	10	9	80
		*108*^b^	*11*	*31*	*10*	*30*	*80*
TPG	288	195	100	33	10	16	80
		*92*^b^	*100*	*45*	*10*	*12*	*80*
DPG-d_10_	222	124	100	31	10	15	80

a Abbreviations: Q1, precursor ion; Q3, product ion; DP, declustering potential; EP, entrance potential; CE, collision energy; and CXP, collision cell exit potential.

b Italicized transitions indicate the qualitative ions monitored.

### 2.5 Method Validation

Calibration curves for all analytes were prepared in both solvent (i.e., MeOH) and urine matrix fortified at concentrations of 0.05, 0.1, 0.2, 0.5, 1, 2, 5, 10, 20, 50, and 100 ng/mL, along with 10 ng/mL internal standard (DPG-d_10_). Matrix-matched calibration curves were also constructed by spiking target analytes (0.05–100 ng/mL) into synthetic urine.

The matrix effect (ME) was evaluated as the percentage of signal enhancement or suppression, using the following equation
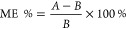
1where *A* and *B* are the slopes of the calibration curves of the analytes in the synthetic urine matrix and solvent (i.e., MeOH), respectively.

The absolute recoveries of target analytes were calculated by comparing analyte peak area in urine samples spiked before and after SPE, using the following equation
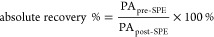
2where PA_pre-SPE_ and PA_post-SPE_ refer to the peak area of an analyte in urine samples fortified with target analytes (at 10 ng/mL) before and after SPE, respectively.

The sensitivity of the method was determined as method limits of detection (LOD) and quantification (LOQ). Six pooled human urine samples were fortified with target analytes at 1 ng/mL, a concentration that generated peaks with signal-to-noise ratio (S/N) values of 40, 93, and 587 for DPG, DTG, and TPG, respectively. LOD and LOQ were estimated as 3 and 10 times, respectively, the standard deviation (SD) of the concentrations measured in human urine fortified at 1 ng/mL.

The method accuracy was assessed based on a spike-recovery experiment performed at low (1 ng/mL), medium (5 and 10 ng/mL), and high (20 ng/mL) concentrations. The precision/trueness of the method was evaluated through intra-day and inter-day variations, which were calculated as the coefficient of variation (CV %) of the measured concentrations in pooled human urine fortified at 1, 5, 10, and 20 ng/mL. Inter-day variations were calculated from the repeated measurement of the fortified urine samples on three different days.

The stability of the analytes in urine was assessed through variations in concentrations (1) in fortified urine samples after three freeze–thaw cycles; (2) holding fortified urine at room temperature (22 °C) overnight; and (3) holding urine extract at room temperature overnight. These experiments were conducted at three fortification levels: low (1 ng/mL), medium (5 ng/mL), and high (20 ng/mL).

### 2.6 Quality Assurance and Quality Control

Quality control (QC) samples included procedural blanks (using LC–MS grade water instead of urine), matrix blanks (pooled human urine), and matrix spikes (pooled human urine fortified with target analytes at 1, 5, 10, and 20 ng/mL). The following QC criteria were set for all analytes: (1) relative to standard solution, deviation in retention time of analytes in urine samples should be <1%; (2) deviation in the ratio of the two MRM transitions for each analyte in urine samples should be <20%; and (3) spike-recoveries for all analytes should be in the range of 70–130%. Furthermore, a solvent blank sample (i.e., MeOH) was injected into LC–MS/MS after every 10 samples to monitor the carryover of target analytes between samples. The measured concentrations of all analytes in procedural blanks and solvent blanks were below the LOD. A mid-point calibration standard (10 ng/mL) was injected after every 20 samples to monitor the stability of the instrumental response to target analytes over time. The instrumental stability was considered acceptable if the deviation in the measured concentrations for all analytes was <10%.

## 3 Results and Discussion

### 3.1 Chromatography and Mass Spectrometry

**Figure 2:**
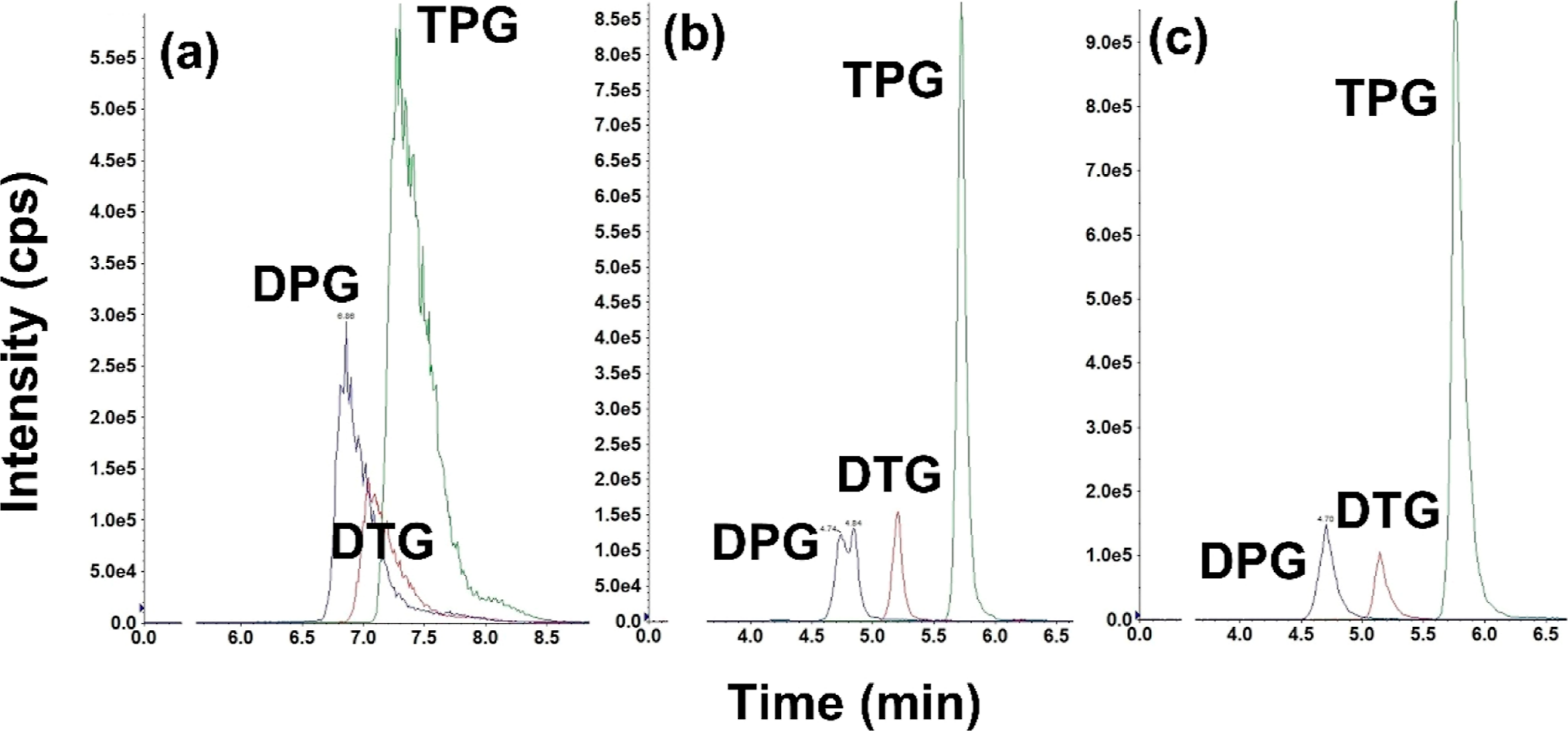
LC–MS/MS chromatograms of DPG, DTG, and TPG in (a) mobile phase A: water; B: MeOH. (b) Mobile phase A: water containing 0.1% formic acid; B: MeOH containing 0.1% formic acid. (c) Mobile phase A: 5 mM ammonium formate containing 0.1% formic acid; B: MeOH containing 0.1% formic acid. Analyte concentrations were 10 ng/mL; and the injection volume was 2 μL.

**Figure 3:**
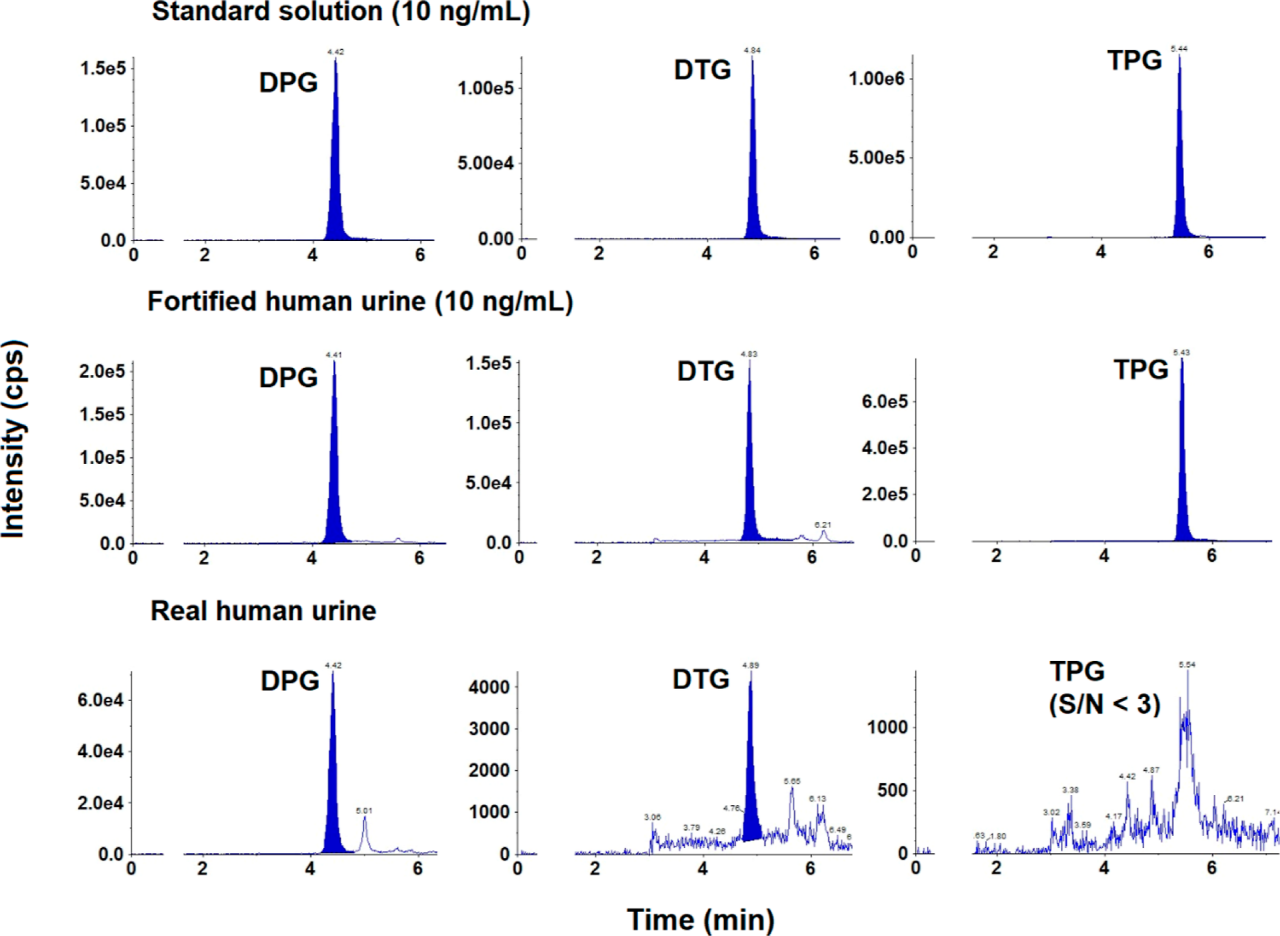
Representative LC–MS/MS chromatograms of DPG, DTG, and TPG in standard solution (10 ng/mL), human urine fortified with all analytes at 10 ng/mL, and real human urine samples. The injection volume was 2 μL.

Given the non-polar properties of DPG, DTG, and TPG, reversed-phase columns (e.g., C18 columns) were considered appropriate for their chromatographic separation.^18^ First, we used an Ultra AQ C18 reversed-phase column (3 μm, 100 × 2.1 mm; Restek, Bellefonte, PA, USA) with water and MeOH as mobile phases. However, all three analytes exhibited poorly resolved, broad, and tailing peaks (Figure 2a). This was probably due to their strong affinities to C18 sorbents (log K*ow* of the analytes: 2.78–5.04; http://www.chemspider.com/). Addition of 0.1% formic acid in mobile phases improved the peak shape and chromatographic resolution (Figure 2b), which can be explained by the loss of electrons and become positively charged in the acidic environment; therefore, they exhibit increased hydrophilicity. Nevertheless, DPG exhibited peak splitting, but the addition of 5 mM ammonium formate as a buffer to mobile phase A eliminated peak splitting (Figure 2c). Following the optimization of the mobile gradient, all analytes were baseline separated and exhibited symmetric peaks (Figures 2, 3, and S1). The MRM parameters of the analytes were optimized through direct infusion of a standard solution (100 ng/mL) into the mass spectrometer via a syringe pump, and the optimized parameters are given in Table 1.

### 3.2 Optimization of Sample Extraction and Purification

Reversed-phase SPE cartridges such as Oasis HLB are suitable for purification of extracts in the analysis of non-polar chemicals.^11,15^ Conditioning and washing steps for HLB cartridges were optimized using fortified urine samples. Cartridges were conditioned by sequential elution of 3 mL of MeOH and 3 mL of water. Washing with 3 mL of MeOH/water (5:95, v/v) adequately removed matrix components from urine (Figure 2).

When solvent-based calibration standards were used to quantify concentrations, DPG showed acceptable accuracies (spike-recoveries: 111–118%), whereas DTG (140–156%) and TPG (224–238%) exhibited recoveries above 140% (Table S1). This could be explained by lower polarities of DTG and TPG than that of DPG and the lack of corresponding isotopically labeled internal standards for DTG and TPG (DPG-d_10_ was used as the internal standard for all three target analytes). Nevertheless, elevated recoveries of DTG and TPG were corrected for, by quantification using matrix-matched calibration curves, which yielded acceptable spike-recoveries for all three analytes in the range of 75.3–111% (Table 2). Thus, matrix-matched calibration curves are recommended in the analysis of DPG, DTG, and TPG, when corresponding isotopically labeled internal standards are not available for all target analytes.

**Table 2:** Method Validation Parameters for the Analysis of DPG, DTG, and TPG in Human Urine^a^

	DPG	DTG	TPG
*R* in solvent	0.9999	0.9995	0.9920
Slope in solvent	0.18	0.10	0.63
*R* in urine matrix	0.9999	0.9998	0.9949
Slope in urine matrix	0.15	0.09	0.42
LOD (ng/mL)	0.02	0.02	0.002
LOQ (ng/mL)	0.05	0.05	0.005
absolute recovery %	44.1	49.0	99.2
ME %	–13.6	–15.0	–33.1
Spike-Recovery%, *n* = 3			
1 ng/mL	107 ± 2	101 ± 3	85.7 ± 0.8
5 ng/mL	102 ± 1	101 ± 1	75.3 ± 0.7
10 ng/mL	111 ± 2	108 ± 1	78.4 ± 0.7
20 ng/mL	106 ± 1	104 ± 4	76.5 ± 1.7
Intra-Day CV %, *n* = 3			
1 ng/mL	1.94	2.96	0.91
5 ng/mL	1.11	1.27	0.93
10 ng/mL	2.08	0.93	0.88
20 ng/mL	0.47	3.90	2.26
Inter-Day CV %, *n* = 3			
1 ng/mL	2.79	0.86	1.29
5 ng/mL	1.03	0.66	0.82
10 ng/mL	3.02	2.67	3.31
20 ng/mL	3.76	2.65	2.96
Variation in Concentration % after Three Freeze–Thaw Cycles (Mean ± SD), *n* = 3			
1 ng/mL	–8.27 ± 10.0	–3.71 ± 4.50	–13.5 ± 4.8
5 ng/mL	2.99 ± 1.37	1.21 ± 2.28	0.82 ± 4.58
20 ng/mL	1.05 ± 17.8	0.97 ± 17.6	1.39 ± 20.6
Variation in Concentration % after Holding Urine at Room Temperature Overnight (Mean ± SD), *n* = 3			
1 ng/mL	–10.5 ± 14.6	–8.77 ± 13.9	–19.2 ± 9.6
5 ng/mL	–5.99 ± 2.74	–3.32 ± 1.05	–11.4 ± 2.2
20 ng/mL	–12.3 ± 3.8	–12.2 ± 3.7	–11.3 ± 5.7
Variation in Concentration % after Holding Urine Extract at Room Temperature Overnight (Mean ± SD), *n* = 3			
1 ng/mL	–25.6 ± 3.1	–24.3 ± 2.4	–26.8 ± 1.0
5 ng/mL	–12.4 ± 1.7	–10.7 ± 5.5	–10.7 ± 1.7
20 ng/mL	–2.11 ± 21.4	–6.05 ± 13.0	–1.62 ± 18.0

a Abbreviations: LOD, limit of detection; LOQ, limit of quantification; ME, matrix effect; CV, coefficient of variation; and *R*, regression coefficient.

### 3.3 Method Validation

An 11-point calibration curve was prepared in both solvent and urine matrix at concentrations in the range of 0.05–100 ng/mL. A weighted (1/*x*) linear regression was used to fit calibration curves, which showed excellent linearity for all analytes in both solvent (*R*-values: 0.9920–0.9999) and urine matrix (*R*-values: 0.9949–9.9999). The slopes of solvent-based calibration curves for DPG, DTG, and TPG were 0.18, 0.10, and 0.63, respectively, whereas those of matrix-matched calibration curves were 0.15, 0.09, and 0.42, respectively (Table 2).

The absolute recoveries, calculated based on the comparison of peak areas of analytes in urine fortified with them before and after SPE were used to assess the recovery of each analyte during sample cleanup (Table 2). The absolute recoveries of DPG, DTG, and TPG following passage through HLB cartridges were 44.1, 49.0, and 99.2%, respectively. The higher absolute recovery of TPG than those of DPG and DTG can be explained by its lower polarity and stronger interaction with sorbents in HLB cartridges.

The electrospray ionization–MS is susceptible to the matrix effect, caused by either suppression or enhancement of the analyte response by matrix components. The matrix effect in the range of −20–+20% is considered weak or low. In this study, DPG (ME: −13.6%) and DTG (−15.0%) exhibited weak ion suppression, indicating that the optimized sample cleanup procedure adequately removed the matrix interferences for these two analytes. However, a moderate/medium ion suppression was found for TPG (−33.1%) (Table 2). Use of isotopically labeled TPG as the internal standard would correct for the matrix effect-related inaccuracies in quantification. Isotopically labeled TPG is not commercially available at this time. In the absence of isotopically labeled internal standards, quantification based on matrix-matched calibration curves can correct for matrix effects.

The accuracy of the method was assessed through spike-recovery experiments conducted in triplicate. Analytes were fortified in pooled human urine at four different levels: 1, 5, 10, and 20 ng/mL. Matrix-matched calibration curves were used for quantification. The spike-recoveries of DPG, DTG, and TPG were in the range of 102–111%, 101–108%, and 75.3–85.7%, respectively, with the respective standard deviation (SD) of 1–2%, 1–4%, and 0.7–1.7%. Slightly low recoveries of TPG (75.3–85.7%) (Table 2) may be due to the lack of isotopically labeled internal standard for this analyte.

The intra-day and inter-day variations, calculated as CV, of repeated analysis of fortified human urine at 1, 5, 10, and 20 ng/mL, were used in the assessment of method precision. The intra-day CVs for DPG, DTG, and TPG were in the range of 0.47–2.08%, 0.93–3.90%, and 0.88–2.26%, respectively, whereas the inter-day CVs were in the range of 1.03–3.76%, 0.66–2.67%, and 0.82–3.31%, respectively. These results suggested excellent precision/trueness of the method for all analytes.

Pooled human urine fortified with each analyte at 1 ng/mL was analyzed six times for the calculation of LOD and LOQ. The LODs of DPG, DTG, and TPG were 0.02, 0.02, and 0.002 ng/mL, respectively, and the corresponding LOQs were 0.05, 0.05, and 0.005 ng/mL. Our method has adequate sensitivity to reliably determine concentrations of DPG, DTG, and TPG at parts-per-trillion levels in urine.

The stability of the analytes was evaluated through variations in concentrations after freeze–thaw cycles and holding samples or extracts at room temperature overnight (Table 2). The analyte concentrations were stable following three freeze–thaw cycles, with variation in concentrations of −8.27–2.99%, −3.71–1.21%, and −13.5–1.39% for DPG, DTG, and TPG, respectively. However, holding urine samples or extracts at room temperature overnight resulted in measurable loss in concentrations, especially at the low fortification level. For example, DPG, DTG, and TPG were lost at −25.6%, −24.3%, and −26.8%, respectively, of the original concentrations in the extract of urine fortified at 1 ng/mL and held at room temperature overnight. Further studies are needed to assess the stability of target analytes over a long-term storage (months or years) at −20 °C or −80 °C. Considering that the concentrations of DPG in urine of the general population is low (Table 3), it is recommended that samples are frozen immediately (preferably at −80 °C), and the extracts are injected into LC–MS/MS as soon as possible.

**Table 3:** Concentrations of DPG, DTG, and TPG Measured in 15 Children’s and 20 Adults’ Urine Samples Collected from New York, United States^a^

	DPG (ng/mL)	DTG (ng/mL)	TPG (ng/mL)
Children’s Urine (*n* = 15)			
DF %	73	13	0
mean	0.40	0.29	<LOD
SD	0.86	0.36	<LOD
min	<LOD	<LOD	<LOD
median	0.05	<LOD	<LOD
max	2.94	0.54	<LOD
Adults’ Urine (*n* = 20)			
DF %	20	5	0
mean	0.25	0.04	<LOD
SD	0.37	0.00	<LOD
min	<LOD	<LOD	<LOD
median	<LOD	<LOD	<LOD
max	0.79	0.04	<LOD

a Abbreviations: DF, detection frequency; SD, standard deviation; min, minimum; and max, maximum.

### 3.4 Application of the Method

The optimized method was applied for the determination of DPG, DTG, and TPG in 15 children’s and 20 adults’ urine samples collected from New York, United States. This is the first study to determine the occurrence of DPG, DTG, and TPG in human urine. DPG was found in 73% of children’s urine samples with a median concentration of 0.05 ng/mL (range: <LOD–2.94 ng/mL). In adults’ urine, DPG was found at a lower detection frequency (DF; 20%) and at a concentration range of <LOD–0.79 ng/mL (Table 2). Greater concentrations found in children’s urine may be related to higher dust ingestion rates (e.g., 50 mg/day for children versus 20 mg/day for adults),^18^ which is presumed as the major pathway of human exposure to DPG.^17−19^ A recent study found DPG in maternal and cord serum at median concentrations of 1.7 and 0.35 ng/mL, respectively.^25^ The higher concentrations of DPG in serum than those in urine may be related to its toxico-kinetics. A laboratory animal study reported that DPG is excreted in equal amounts through urine and feces. Furthermore, ∼28% of DPG excreted in urine was present as the parent compound, whereas the remainder was in the form of metabolites.^24^ Nevertheless, metabolites of DPG were not identified, and further studies are warranted on this regard. Our study provides evidence of exposure to DPG and DTG in humans. Representative LC–MS/MS chromatograms of targeted analytes in solvent, fortified urine, and real urine samples are shown in Figure 3.

DTG was found in only 13 and 5%, respectively, of children’s and adults’ urine samples. This indicates relatively low exposure rates to DTG, likely due to its low consumption volume (61 tons in the United States in 2019; https://chemview.epa.gov/chemview/). In addition, TPG was not detected in all samples analyzed in this study (Tables 3 and S2).

A recent study from China reported the occurrence of 6PPD and 6PPD-Q in children’s urine (*n* = 50) at DFs of 60 and 90%, respectively, and at median concentrations of 0.015 and 0.076 ng/mL, respectively.^10^ The DF and concentrations of DPG measured in children’s urine in this study were similar to those of 6PPD and 6PPD-Q. Nevertheless, further studies with larger sample size are needed to measure these rubber additives simultaneously.

In summary, we developed and validated a method for the determination of DPG, DTG, and TPG in human urine using isotope dilution HPLC–MS/MS. Passage of urine samples through a HLB cartridge adequately removed matrix interferences. By use of matrix-matched calibration curves, acceptable accuracies and precisions were obtained for all analytes. Application of the optimized method revealed the occurrence of DPG in children’s and adults’ urine samples. The developed method is suitable for the assessment of exposure to DPG, DTG, and TPG in human populations. Nevertheless, it should be noted that DPG, DTG, and TPG can be metabolized in humans, and further studies are warranted to identify the metabolites of these chemicals in urine.

## Supporting Information Available

Spike-recoveries of all analytes in urine using solvent-based calibration curves; concentrations of DPG, DTG, and TPG measured in each urine sample; and representative chromatograms of DPG, DTG, and TPG in the solvent using the optimized conditions (PDF)
