# Supplementary material for: Determination of 1,3-Diphenylguanidine, 1,3-Di-o-tolylguanidine, and 1,2,3-Triphenylguanidine in Human Urine Using Liquid Chromatography-Tandem Mass Spectrometry
Source: Environ Sci Technol. 2023 Jun 8;57(24):8883–9. doi: 10.1021/acs.est.3c00412 (PMC10286301; doi:10.1021/acs.est.3c00412)
Supplement: Supplementary file 1 — es3c00412_si_001.pdf [file es3c00412_si_001.pdf]

Supporting Information

Determination of 1,3-Diphenylguanidine, 1,3-Di-*o*-tolylguanidine, and 1,2,3-Triphenylguanidine in Human Urine using Liquid Chromatography-Tandem Mass Spectrometry

Zhong-Min Li[a,b] and Kurunthachalam Kannan*[a,b]

[a]Department of Pediatrics, New York University Grossman School of Medicine, New York, NY 10016, United States

[b]Department of Environmental Medicine, New York University Grossman School of Medicine, New York, NY 10016, United States

Corresponding Author

Kurunthachalam Kannan

Address: MSB 6-698, 550 First Avenue, New York, NY 10016, United States

Tel.: (212)-263-1546

E-mail: kurunthachalam.kannan@nyulangone.org

Number of pages: 5

Number of tables: 2

Number of figures: 1

Table of contents

Table S1 Spike-recoveries of 1,3-diphenylguanidine (DPG), 1,3-di-o-tolylguanidine (DTG), and 1,2,3-triphenylguanidine (TPG) in human urine using solvent-based calibration curve. Data are shown as mean ± SD S3

Table S2 Measured concentrations of 1,3-diphenylguanidine (DPG), 1,3-di-*o*-tolylguanidine (DTG), and 1,2,3-triphenylguanidine (TPG) in 15 children's urine and 20 adults' urine samples S4

Fig. S1 Representative chromatograms of the analytes targeted in this study. Concentrations of the analytes were 10 ng/mL. The injection volume was 2 μL S5

Table S1 Spike-recoveries of 1,3-diphenylguanidine (DPG), 1,3-di-*o*-tolylguanidine (DTG), and 1,2,3-triphenylguanidine (TPG) in human urine using solvent-based calibration curve. Data are shown as mean ± SD.

Spike-level (ng/mL)	DPG (%)	DTG (%)	TPG (%)
1	113 ± 2	143 ± 4	224 ± 6
5	111 ± 3	140 ± 6	224 ± 6
10	118 ± 2	151 ± 2	238 ± 4
20	116 ± 2	146 ± 2	236 ± 6

Table S2 Measured concentrations of 1,3-diphenylguanidine (DPG), 1,3-di-*o*-tolylguanidine (DTG), and 1,2,3-triphenylguanidine (TPG) in 15 children's urine and 20 adults' urine samples.

ID	DPG (ng/mL)	DTG (ng/mL)	TPG (ng/mL)
**Children's urine**			
Child-1	0.06	< LOD	< LOD
Child-2	0.04	< LOD	< LOD
Child-3	0.11	< LOD	< LOD
Child-4	0.03	0.03	< LOD
Child-5	< LOD	< LOD	< LOD
Child-6	0.02	< LOD	< LOD
Child-7	2.94	< LOD	< LOD
Child-8	0.52	< LOD	< LOD
Child-9	< LOD	< LOD	< LOD
Child-10	0.29	< LOD	< LOD
Child-11	0.05	0.54	< LOD
Child-12	0.29	< LOD	< LOD
Child-13	0.06	< LOD	< LOD
Child-14	< LOD	< LOD	< LOD
Child-15	< LOD	< LOD	< LOD
**Adults' urine**			
Adult-1	< LOD	< LOD	< LOD
Adult-2	0.03	< LOD	< LOD
Adult-3	< LOD	< LOD	< LOD
Adult-4	< LOD	< LOD	< LOD
Adult-5	0.79	< LOD	< LOD
Adult-6	< LOD	< LOD	< LOD
Adult-7	< LOD	< LOD	< LOD
Adult-8	< LOD	< LOD	< LOD
Adult-9	< LOD	< LOD	< LOD
Adult-10	< LOD	< LOD	< LOD
Adult-11	< LOD	< LOD	< LOD
Adult-12	< LOD	< LOD	< LOD
Adult-13	< LOD	< LOD	< LOD
Adult-14	0.02	< LOD	< LOD
Adult-15	< LOD	< LOD	< LOD
Adult-16	0.14	< LOD	< LOD
Adult-17	< LOD	< LOD	< LOD
Adult-18	< LOD	< LOD	< LOD
Adult-19	< LOD	< LOD	< LOD
Adult-20	< LOD	0.04	< LOD

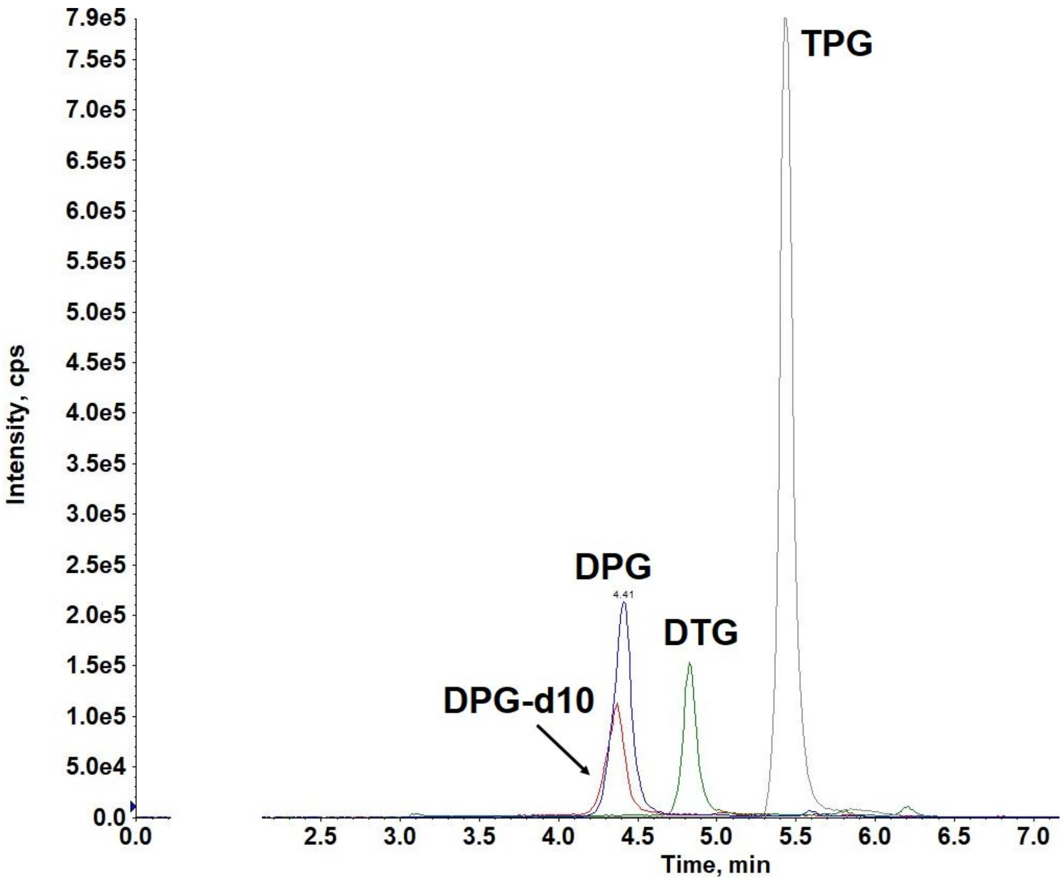

Fig. S1 Representative chromatograms of the analytes targeted in this study. Concentrations of the analytes were 10 ng/mL. The injection volume was 2 μL.
